# Severe hypophosphatemia in the critically ill: are we replacing enough?

**DOI:** 10.1186/2197-425X-3-S1-A913

**Published:** 2015-10-01

**Authors:** J Millwood Hargrave, K Agarwal, A Ahmed, C-H Toh, C Downey, ID Welters

**Affiliations:** Royal Liverpool University Hospital, Intensive Care Unit, Liverpool, United Kingdom; University of Liverpool, Institute of Ageing and Chronic Disease, Liverpool, United Kingdom; Royal Liverpool University Hospital, Department of Haematology, Liverpool, United Kingdom; University of Liverpool, Institute of Infection and Global Health, Liverpool, United Kingdom

## Introduction

Severe hypophosphatemia is frequently seen in the critically ill, and its presence is associated with significant morbidity. Although phosphate correction is commonly performed in a critical care environment, the optimal amount of phosphate supplementation and the role of serum phosphate levels in the early days following admission remains uncertain.

## Objectives

We sought to establish the course of severe hypophosphatemia in the early days of critical illness (Day 1 - 7) and to ascertain whether phosphate supplementation delivered to severely hypophosphatemic patients led to appropriate rise in serum phosphate concentrations.

## Methods

Patient data over seven days following admission was retrospectively collected for 1,038 admissions to critical care units of a tertiary hospital. 22 patients with severe hypophosphatemia (Serum phosphate < 0.4 mmol/l) were identified from this data set. In these patients, demographic and clinical data as well as time course of serum phosphate concentrations and total oral and parenteral phosphate supplementation were recorded. The clinical course for serum phosphate levels was observed during the first seven days following admission. A local protocol for phosphate replacement is in place which suggests administration of up to 40 mmol/day in severe hypophosphatemia. All patients received phosphate supplementation within the seven days following admission, and the daily supplementation given was compared to the recommended phosphate supplementation as per local protocol.

## Results

22 patients were eligible for inclusion. The mean age was 48.2 ± 15.5 years, the mean APACHE II score 18.9 ± 7.0, and 50% of patients were female. Mean serum phosphate levels normalised from admission (0.32 mmol/l ± 0.06) to day five (0.90 mmol/l ± 0.22) in the majority of patients (73.7%).

There was a significant difference between the phosphate supplementation severely hypophosphatemic patients received (7.23 ± 4.85 mmol) compared to the recommended phosphate replacement (14.91 mmol) as per local protocol, over the first seven days following admission.

## Conclusions

Phosphate supplementation in severe hypophosphatemia is less than recommended. As a consequence, normalisation of phosphate levels was achieved only after 5 days of Intensive Care and only in about three quarter of severely hypophosphatemic patients. This may reflect the need for more aggressive phosphate supplementation in our critical care unit.

## Grant Acknowledgment

We have read and understood ESICM policy on declaration of grants and declare that we have received no grants for this project.
